# Evaluation of Quantitative Computed Tomography Indices in Patients with Pneumonia and Acute Respiratory Failure in the Intensive Care Unit (ICU)

**DOI:** 10.3390/diagnostics16050685

**Published:** 2026-02-26

**Authors:** Volkan Alparslan, Özgür Çakır, Özlem Güler, Yusuf Altıntaş, Pınar Kartal Köse, Sibel Balci, Ahmet Yalnız, Nur Baykara, Alparslan Kuş

**Affiliations:** 1 Department of Anaesthesiology and Reanimation, University of Kocaeli, Kocaeli 41380, Turkey; 2 Department of Radiology, University of Kocaeli, Kocaeli 41380, Turkey; 3 Department of Infectious Diseases and Clinical Microbiology, University of Kocaeli, Kocaeli 41380, Turkey; 4 Department of Biostatistics and Medical Informatics University of Kocaeli, Kocaeli 41380, Turkey

**Keywords:** acute respiratory failure, intensive care unit, pneumonia, quantitative computed tomography

## Abstract

**Background:** In this study, we aimed to explore the relationship between quantitative indices derived from computed tomography (CT) attenuation histograms and disease prognosis in patients with pneumonia and acute respiratory failure. We also sought to assess the effectiveness of these parameters as clinical prognostic markers. **Methods:** CT images of patients with pneumonia and acute respiratory failure were analyzed using Vitrea^®^ Advanced Visualization software. The analyzed quantitative CT (qCT) indices included mean lung Hounsfield unit (HU) and density-based volume measurements, specifically low-, medium-, and high-density volume (LDV, MDV, and HDV). Comparative analyses were performed to examine the differences in the volume density between the lungs bilaterally; these were accompanied by regional analyses and density indices. All indices were calculated using previously defined and validated Hounsfield unit (HU) thresholds, which helped to ensure accurate and consistent quantitative measurements and facilitated a more robust evaluation of the prognostic potential of qCT parameters. **Results:** Quantitative CT indices proved to have significant prognostic value in predicting mortality. In multivariable analysis, Difference for Lung HDV > 193 mL emerged as an independent risk factor (aOR: 4.29, *p* = 0.041). The prognostic significance was especially evident in patients with unilateral dominant pneumonia, where Difference for Lung MDV >219 mL (aOR: 9.30, *p* = 0.03) and Difference for Lung HDV > 193 mL (aOR: 10.85, *p* = 0.02) emerged as strong independent predictors of mortality. In this subgroup, lung volume differences demonstrated the strongest diagnostic performance (AUC: 0.808, 95% CI: 0.667–0.908, *p* < 0.001). **Conclusions:** Clinical outcomes are associated with quantitative CT-derived lung volume and density difference indices. Inter-lung differences in Lung MDV and Lung HDV are linked to mortality and may provide additional prognostic information beyond conventional imaging methods. Prospective studies should be conducted to validate these findings, and caution should be exercised during their interpretation.

## 1. Introduction

Acute respiratory failure, which occurs when the lungs are unable to sufficiently oxygenate arterial blood and/or prevent carbon dioxide retention, is one of the leading causes of admission to the intensive care unit (ICU) [1,2]. Although the generally accepted values are PaO2 < 60 mmHg, PaO2/FiO2 < 300 mmHg, and/or PaCO2 > 50 mmHg, it is essential to evaluate the full clinical picture and individual patient factors prior to establishing a diagnosis [3]. Acute respiratory failure has several potential causes; the most common include pneumonia, acute respiratory distress syndrome (ARDS), cardiogenic edema, and sepsis [4]. Pneumonia leads to changes in respiratory mechanics and impairs the gas exchange function of the lungs, as well as the ventilation–perfusion balance and spirometric values. Patients with pneumonia who require mechanical ventilation are at increased risk of mortality and morbidity [5].

Computed tomography (CT) is typically a fundamental component of the diagnostic approach in cases of pneumonia accompanied by acute respiratory failure. Lung parenchyma can be evaluated using CT, but tests such as spirometry are needed to detect functional and volumetric changes. However, conducting spirometric testing in critically ill patients is challenging and is often impractical in clinical practice. Technological advances in Quantitative Computed Tomography (qCT) measurements enable clinicians to evaluate lung volumetric status without spirometry [6]. Lung volumetric measurements are essential data for clinicians, facilitating diagnosis and assessment of severity, treatment planning, and the prediction of outcomes for lung diseases [7]. Numerous studies have demonstrated a strong correlation between qCT and physiological measurements during follow-up of patients with interstitial lung disease, chronic obstructive pulmonary disease, small airway diseases, SARS-CoV-2 pneumonia, etc. [5,8,9,10,11].

Studies have also revealed that qCT measurements correlate significantly with disease prognosis and spirometric parameters in chronic lung diseases [10,11,12]. In critically ill patients with acute respiratory failure, qCT analysis can provide important information about disease severity and prognosis, helping clinicians to optimize treatment strategies in the early stages of disease [13].

In this study, we investigated the relationship between quantitative parameters from CT attenuation histograms and disease prognosis in patients with pneumonia and acute respiratory failure and assessed qCT parameters’ effectiveness as clinical prognostic markers. We analyzed the mean lung Hounsfield unit (HU) and density-based volume measurements of low-, medium-, and high-density volume (LDV, MDV, and HDV). We also conducted comparative analyses of LDV, MDV, and HDV between bilateral lungs, alongside regional analyses and density indices. HU thresholds, as defined and validated in the literature, were used to calculate these parameters [14].

## 2. Methods

### 2.1. Patient Selection

We retrospectively analyzed data from the records of the Kocaeli University Faculty of Medicine. Our study included 89 adult patients (aged ≥ 18 years) who were admitted to the ICU with pneumonia and acute respiratory failure between 2017 and 2024. The patients underwent CT upon arrival and were not intubated at the time of ICU admission. All chest CT scans were performed according to routine clinical indications.

CT scans performed for diagnostic purposes in the emergency department or hospital wards at the time of ICU admission were classed as screening CTs.

The exclusion criteria were as follows: (1) non-infectious causes of acute respiratory failure, (2) being positive for COVID-19, (3) pulmonary malignancy, (4) chronic lung disease, and (5) requiring intubation within the first 10 days of ICU admission for reasons other than acute respiratory failure (e.g., airway obstruction or neurological deterioration). Patients who were already intubated upon ICU admission were also excluded. Our rationale behind these exclusions was to reduce heterogeneity and confounding factors.

In the first stage, to evaluate the short-term outcomes, 89 patients were divided into two groups: survivors and those who died within the first 14 days.

In the second stage, CT evaluation was performed according to the degree of lung parenchymal involvement, and any discrepancies were resolved based on consensus between two radiologists. Predominant involvement was defined as the presence of parenchymal disease in one lung that was assessed visually on axial CT sections, with the condition being present in more than 50% of the lung. Subsequent to the identification of subgroups in which lung involvement predominantly occurred in the right or left lung, we compared survivors and non-surviving patients within these groups. This second-stage analysis was exploratory in nature and was intended to generate hypotheses regarding the impact of lateralized disease patterns on outcomes.

This study was approved by the Ethics Committee of the Kocaeli University Faculty of Medicine in Kocaeli, Turkey, with reference number KU GOKAEK-2024/07.45 (project number: 2024/216). The trial was registered with ClinicalTrials.gov under the trial registration number NCT06651931 on 15 October 2024.

### 2.2. Quantification of Images and Measurement of Quantitative CT (qCT)

Baseline CT scans from patients diagnosed with pneumonia and acute respiratory failure who were admitted to the intensive care unit between 2017 and 2024 were retrieved from the radiology archive. Patients who were filmed in the supine position and were able to fully inspire during filming were included in the study. All scans were performed using a 320-slice multidetector CT scanner (Aquilion ONE^TM^, Toshiba Medical Systems, Tokyo, Japan) using the following scan parameters: 250 mAs, 120 kV, pitch 1, 16 × 1 mm collimation, 1 mm reconstruction interval, and 1 mm reconstruction slice thickness. The sensitivity increased as the collimation and slice thickness decreased [15]. Scans with inadequate image quality or significant motion artifacts were excluded from the quantitative analysis (n = 3), and the remaining patients were included in the final quantitative analysis.

The lung parenchyma was analyzed with a window width of 1600 Hounsfield units (HU) and a level of −500 HU. Scans were analyzed using Vitrea Advanced Visualization software (Canon Group, Minnetonka, MN, USA) for image quantification. The HU thresholds for LDV (−1024 to −920 HU), MDV (−920 to −720 HU), and HDV (−720 to 0 HU) were selected according to the default settings of Vitrea CT Lung Density Analysis software version 7.12.3 [16] and were consistent with prior quantitative CT literature evaluating emphysematous, poorly aerated, and normally aerated lung tissue compartments.

Quantitative CT (qCT) indices were analyzed according to five main categories.

1. Lung density measurements: Low-, medium-, and high-density volume for the right and left lungs, and their total values.
2. Volume measurements: These included the right and left lungs, total lung volume, and airway volume. The total lung volume (TLV) was equivalent to the total volume of lung voxels (LDV + MDV + HDV) within the range of −1024 to 0 HU.
3. Density indices: Right/left low-density index (LDI%), total LDI and 15th percentile lung density (PD15) values. LDI was defined as the ratio of the lung volume below the low-density threshold to the total lung parenchymal volume, including all voxels below the upper density threshold. LDI% was calculated as follows: LDI% = low volume/(low volume + medium volume). The 15th percentile point, defined in HU, was a sensitive densitometric index. The PD15 threshold represents the point at which 15% of the lowest-density lung voxels were distributed [11,12].
4. Regional analyses: Upper/lower lung LDI and upper/lower ratio.
5. Hounsfield unit measurements: The mean HU of the right and left lungs and the mean HU of the airways.

Density-based measurements (LDV, MDV, and HDV) demonstrated the volumetric distribution of lung tissue density and provided key information for evaluating disease severity. Regional analyses were crucial for identifying disease distribution patterns and highlighting the differences between upper and lower lung involvement. Meanwhile, HU measurements provided a quantitative method for assessing tissue density and objectively determining the extent of pathological changes.

During image analysis, the main airways, blood vessels, airway bifurcations, lungs, and lobes were automatically identified. However, the blood vessels and airways up to the subsegmental level were eliminated from the quantification process [17]. Anatomical structures were excluded from the analysis, which could lead to errors in the lung parenchyma density assessment.

The differences in the qCT indices between the two lungs (right and left) were also calculated.

Three different density ranges were used for quantitative evaluation.

- LDV: −1024 to −920 HU.
- MDV: −920 to −720 HU.
- HDV: −720 to 0 HU.

### 2.3. Statistical Analysis

Statistical analyses were performed using IBM SPSS 29.0 (IBM Corp., Armonk, NY, USA) and MedCalc 14 (MedCalc Software, Ostend, Belgium). The normality assumption was assessed using the Kolmogorov–Smirnov and Shapiro–Wilk tests. Normally distributed continuous variables are expressed as the mean ± standard deviation, while non-normally distributed continuous variables are expressed as the median and interquartile range (IQR). Categorical variables are reported as counts and percentages. Comparisons between the surviving and deceased patients with respect to continuous variables, such as age, time to intubation, ICU length of stay, ventilator days, hospital length, APACHE II, SOFA, and quantitative CT indices, were performed using either the independent samples *t*-test or the Mann–Whitney U test, while the categorical variables of the two groups, such as sex, need for IMV, comorbidities, and care dependency, were assessed using the Chi-square test. Multivariable analysis was conducted using binary logistic regression. Receiver operating characteristic (ROC) analysis was performed to compute the area under the curve (AUC), sensitivity, specificity, and cut-off values. Comparisons between AUCs were conducted using DeLong’s test. A *p*-value of <0.05 was considered statistically significant.

## 3. Results

This study included 89 patients with pneumonia and acute respiratory failure. None of the patients were excluded. Prognostic indicators were assessed in two phases: first for the entire cohort and then for the subgroup of patients with predominantly unilateral pneumonic involvement.

All the patient characteristics recorded during the first stage are presented in Supplementary Table S1. The etiologic agents of pneumonia are shown in Supplementary Table S2, the laboratory parameters are presented in Supplementary Table S3, and the quantitative CT indices for the first stage are listed in Supplementary Table S4.

The results of the multivariable and univariable analyses are presented in Table 1.

In the second stage, patients with predominant unilateral pneumonia were categorized as survivors or deceased. Their baseline characteristics are listed in Table 2, their quantitative CT indices can be found in Table 3, and the results of the univariable and multivariable analyses are provided in Table 4.

The etiologic agents of pneumonia are detailed in Supplementary Table S5, and the laboratory parameters are presented in Supplementary Table S6.

Univariable and multivariable analyses of patients with predominantly right- or left-sided pneumonia are presented in Table 4 for the second stage. Owing to the high correlation between certain parameters, not all qCT parameters could be included in the multivariable analysis. A strong correlation between ICU and hospital length of stay was detected, so only the former was included in the multivariable analysis.

ROC analysis was performed to determine the cut-off values for the effects of HDV, LDV, MDV, and volume differences on mortality. The analysis was conducted in groups stratified by the predominant side, considering both overall patient outcomes and, more specifically, the outcomes of patients with distinct unilateral pneumonia (Table 5). The threshold values were as follows: >45 mL for Difference for Lung LDV, >193 mL for Difference for Lung HDV, and >246 mL for Difference for Lung Volume parameters (Table 5).

**Figure 1:**
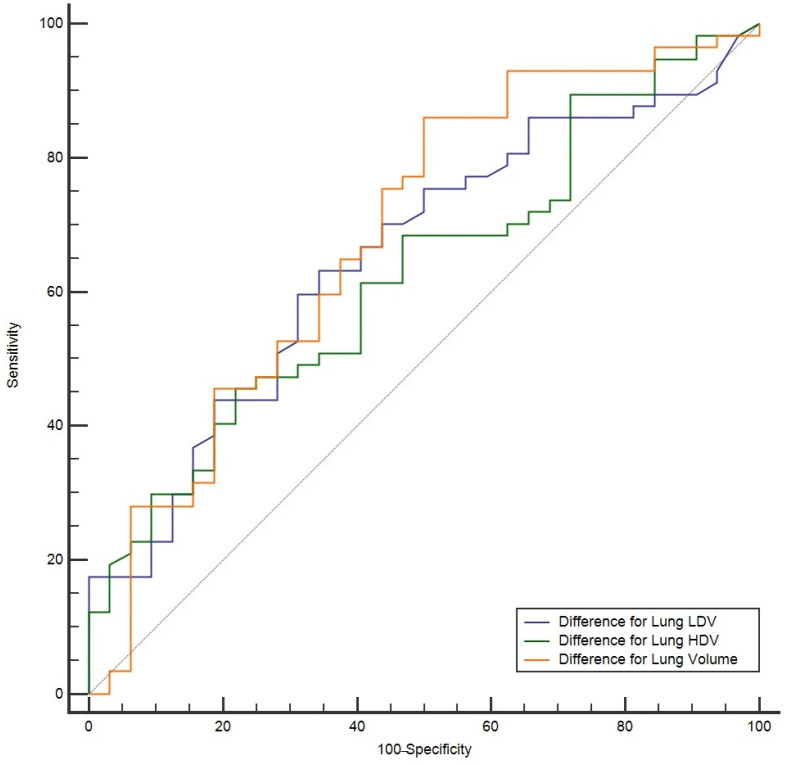
The AUROC curve for Stage I. Sensitivity and specificity are expressed as percentages (%), with the horizontal axis representing specificity (%).

In the first stage, the ROC curves in Figure 1, based on the analysis of the entire cohort, illustrate the sensitivity and specificity values of the Lung LDV difference, Lung HDV difference, and lung volume difference parameters.

**Figure 2:**
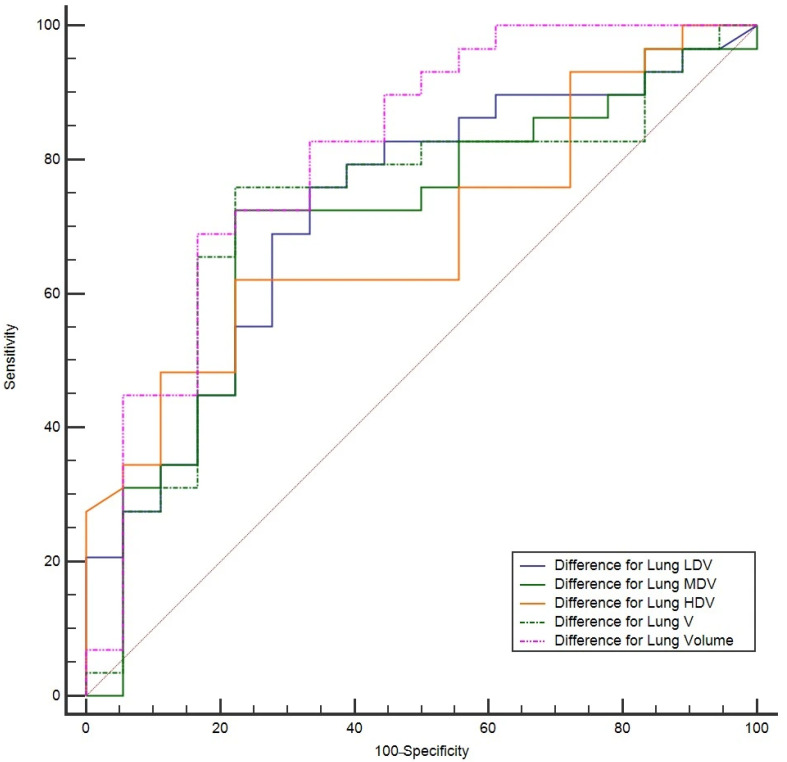
The AUROC curve for Stage II. Sensitivity and specificity are expressed as percentages (%), with the horizontal axis representing 100 − specificity (%).

In the second stage, the threshold values were established as follows: >550 mL for Difference for Lung Volume pa, >45 mL for Difference for Lung LDV, >219 mL for Difference for Lung MDV, >193 mL for Difference for Lung HDV, and >264 mL for Difference for Lung V parameters (Table 5). The ROC curves obtained for unilateral lung parameters are presented in Figure 2.

To assess incremental prognostic value, we compared models with and without the Lung HDV (difference) parameter. Lung HDV (difference) was selected for model expansion because it was the only qCT parameter that remained an independent predictor of mortality in the multivariable analysis for Stage 1. Other qCT variables were not included in order to avoid model overfitting. A comparative ROC analysis using DeLong’s test was performed between Model 1 (SOFA + APACHE II) and Model 2 (SOFA + APACHE II + Lung HDV (difference)). Model 1 demonstrated an AUC of 0.758 (95% CI: 0.655–0.842). After inclusion of the Lung HDV difference parameter, Model 2 showed an AUC of 0.800 (95% CI: 0.702–0.878). Although Model 2 yielded a numerically higher AUC, the difference between the two models was not statistically significant according to DeLong’s test (*p* = 0.167).

**Figure 3:**
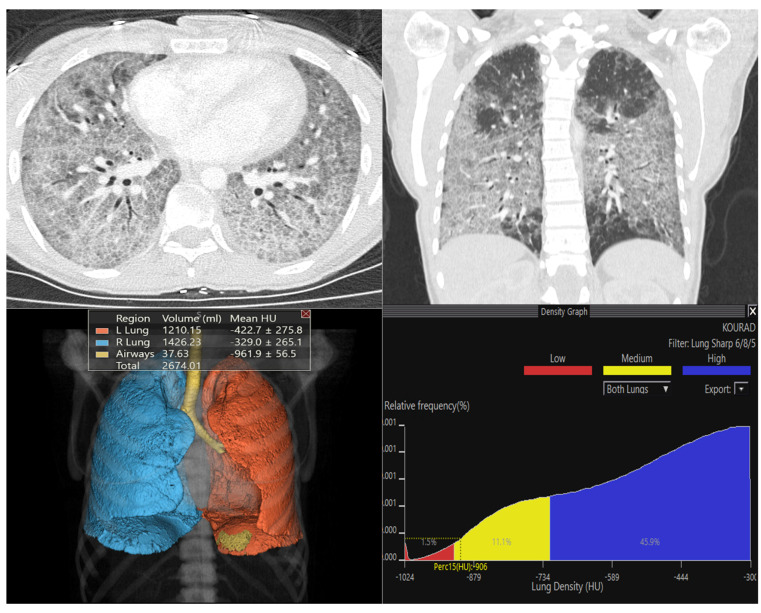
Quantitative analysis of a case presenting with bilateral symmetric high-density parenchymal involvement. The upper panel reveals diffuse, symmetric ground-glass opacities and consolidation areas in both lungs on axial and coronal CT sections. The volumetric analysis in the lower left quadrant demonstrates a marked increase in mean parenchymal density (right: −329 HU; left: −422 HU). The histogram analysis in the lower-right quadrant shows a pathological shift of the density curve to the right (toward higher density). It is observed that normal aerated lung tissue has been largely replaced by high-attenuation (consolidated) areas, represented by the blue zone in the graph, which accounts for 45.9% of the total volume.

**Figure 4:**
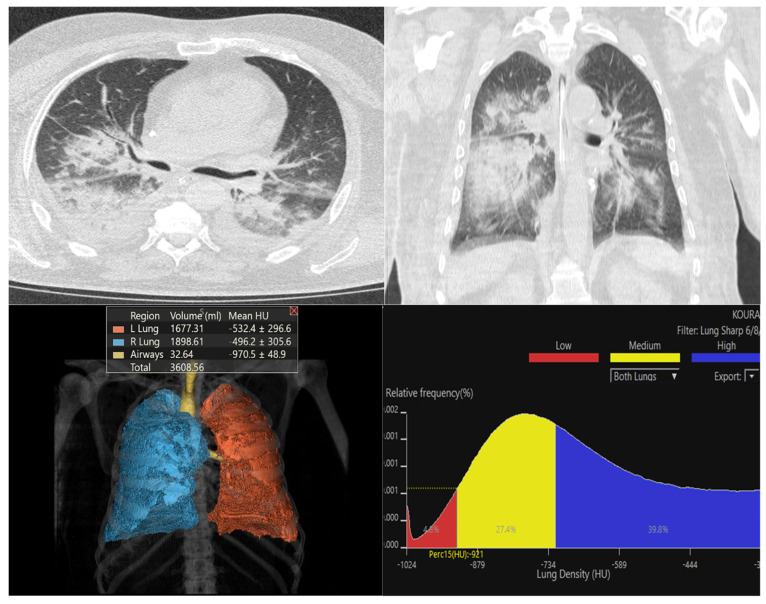
Quantitative analysis of a case presenting with bilateral but right-dominant asymmetric parenchymal involvement. The CT sections in the upper panel reveal diffuse ground-glass opacities and patchy consolidation in both lungs, with the pathology being visibly denser in the right hemithorax. Quantitative data in the lower left quadrant corroborate this visual asymmetry; the mean density of the right lung (mean HU: −496.2 HU) is markedly higher than that of the left lung (mean HU: −532.4 HU). The histogram analysis in the lower right shows a heterogeneous density distribution, where high-density areas (blue, 39.8%) and medium-density transition zones (yellow, 27.4%) predominate over normal parenchyma.

**Figure 5:**
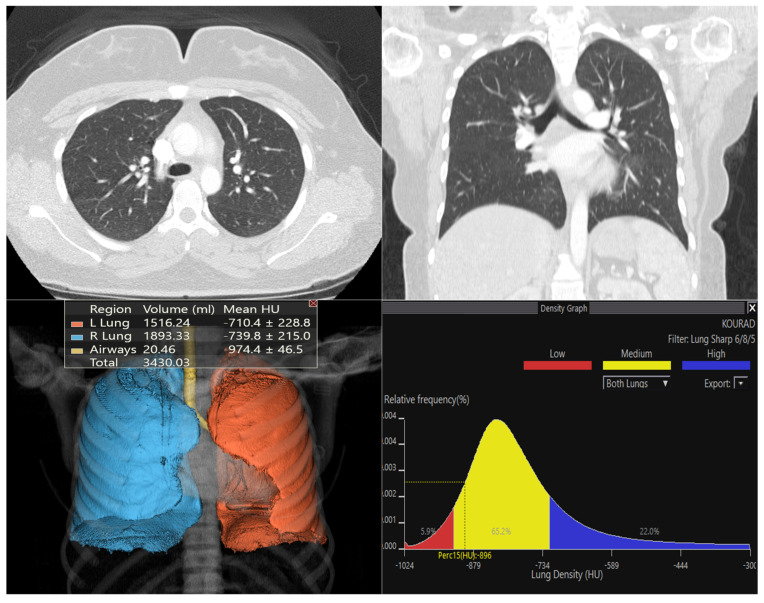
Quantitative analysis of a control subject with no respiratory pathology. The upper panel shows normal parenchymal aeration and vascular structures on axial and coronal computed tomography (CT) sections. The lower left quadrant displays 3-dimensional (3D) volumetric segmentation, where the right (blue) and left (orange) lung volumes were calculated separately (right: ~1893 mL; left: ~1516 mL). The density histogram analysis in the lower right quadrant reveals a normal Gaussian distribution, with mean parenchymal attenuation values ranging from −710 HU to −740 HU, showing no significant deviation indicative of fibrosis (high density) or air trapping (low density).

Figure 3, Figure 4 and Figure 5 show representative Vitrea outputs (CT images, 3D segmentation, and attenuation histograms) for symmetric, asymmetric, and normal lungs.

## 4. Discussion

In this study, we investigated the prognostic value of quantitative CT parameters in patients with pneumonia who presented with acute respiratory failure in a two-stage setting. In the univariable analysis, a Difference for Lung LDV >45 mL, a Difference for Lung HDV >193 mL, and a Difference for Lung Volume >246.3 mL were found to be significantly associated with mortality (OR, 3.27; *p* = 0.010; OR, 2.99; *p* = 0.029; OR, 6.13; *p* < 0.001, respectively). In the multivariable analysis, only Difference for Lung HDV >193 mL was found to be an independent risk factor (aOR: 4.29, *p* = 0.041). In the second stage of our analysis, stronger associations were observed in the subgroup of patients with a clearly predominant side of pneumonia. In this group, Difference for Lung MDV >219 mL (aOR: 9.30, *p* = 0.03) and Difference for Lung HDV >193 mL (aOR, 10.85; *p* = 0.02) were associated with mortality. ROC analyses also supported these findings and showed that quantitative CT parameters had a better diagnostic performance in patients with unilateral dominant pneumonia. Specifically, in the unilateral group, the lung volume (difference) parameter had the highest predictive value with an AUC of 0.808 (95% CI: 0.667–0.908, *p* < 0.001). This high AUC value indicates that changes in lung volume may be clinically significant. Similarly, Lung LDV (difference), Lung MDV (difference), and Lung HDV (difference) demonstrate limited yet significant diagnostic performance, characterized by varying combinations of sensitivity and specificity (e.g., AUC = 0.703, *p* = 0.012; AUC = 0.691, *p* = 0.014). These findings suggest that quantitative CT parameters could be reliable tools for prognostic evaluation, especially in patients with a distinctly predominant side of pneumonia. Changes in these parameters may reflect the extent of the lung tissue damage.

We observed that quantitative CT parameters, particularly Lung HDV (difference), retained independent predictive value in the first stage. Although these findings suggest that qCT metrics may provide prognostic information beyond conventional assessment, this interpretation should be made cautiously. To further evaluate incremental prognostic value, we compared models with and without the Lung HDV difference parameter. Although the addition of this parameter increased the AUC from 0.758 to 0.800, this improvement did not reach statistical significance (*p* = 0.1672). Therefore, the added prognostic value of qCT parameters beyond established clinical scores (SOFA and APACHE II) has not been definitively demonstrated in the present study.

The second-stage analysis was exploratory in nature and aimed to investigate the prognostic relevance of clinically recognizable unilateral disease patterns, which were initially identified by visual assessment and subsequently quantified using objective qCT indices.

Kang et al. [18] demonstrated that quantitative CT-derived lung metrics provide prognostic information in patients with COVID-19 pneumonia, showing strong associations between volumetric density scores and clinical deterioration; however, inter-lung differences and lung asymmetry have not been the primary focus of prior prognostic analyses in viral pneumonia. In this context, our study expands upon the existing literature by specifically addressing inter-lung asymmetry and demonstrating that differences in lung volume and density parameters carry independent prognostic value in patients with pneumonia and acute respiratory failure.

Quantitative CT findings have provided important information for the evaluation of fibrotic lung diseases. Quantitative CT analysis has been identified as a promising tool for evaluating fibrotic lung diseases. However, standardization in clinical practice is necessary to ensure accuracy and reliability. Our findings support this view and demonstrate the potential application of quantitative measurements in disease prognosis assessments. However, before quantitative CT analysis can be integrated into routine clinical practice, several challenges must be addressed. As Egashira and Raghu noted, it is critical to standardize measurement protocols and validate software tools [19]. Combined visual and quantitative CT analyses offer significant advantages in identifying COPD subtypes and predicting prognosis [20]. Our study demonstrated the efficacy of this approach in a clinical setting.

Quantitative CT analysis is a valuable tool for assessing emphysema patterns in COPD patients. Lynch et al. [21] demonstrated that quantitative measurements are useful for differentiating between paraseptal and centrilobular emphysema. Our study also revealed significant changes in the HDV, MDV, and Total Lung Volume on the affected side.

The integration of visual and quantitative CT features is essential for understanding the heterogeneous nature of COPD. Recent studies have demonstrated that this combined approach facilitates the identification of ten distinct subtypes [22], which may help to predict the prognosis and treatment response. Integrating artificial intelligence-assisted analyses with traditional quantitative measures offers new perspectives for evaluating COPD [23].

These data demonstrate the potential prognostic value of qCT measurements when the lungs are affected by both inflammatory and other pathological processes. There is notable divergence between HDV and MDV, particularly in cases of acute conditions such as pneumonia, which may serve as a predictor of mortality risk.

Quantitative CT analysis enables objective assessment of lung attenuation patterns beyond visual interpretation. By providing standardized metrics of aeration loss and parenchymal involvement, qCT may offer additional prognostic information beyond conventional CT findings and routine clinical parameters.

Nevertheless, this study had several limitations. Firstly, the sample size was limited, and the data were derived from a single center with a retrospective design. Secondly, the selection of patients within the context of critically ill ICU populations is inherently challenging. The third predominant involvement was defined in a semi-quantitative manner based on visual assessment, which may have introduced subjectivity despite the consensus between the two radiologists. Furthermore, a direct comparison of qCT indices with radiographic markers, such as consolidation scores, was not feasible, as these variables were inconsistently documented in the dataset. Our findings suggest that there is an association between qCT parameters and short-term mortality. Nevertheless, the value of these markers in relation to other potential clinical markers has yet to be determined and requires validation in a prospective study. Collectively, these limitations underscore the need for larger multicenter studies to validate our findings and elucidate the clinical applicability of quantitative CT indices.

## 5. Conclusions

In this study, quantitative CT-derived lung dose–volume difference parameters, specifically Lung MDV and Lung HDV differences, were associated with mortality in patients with pneumonia and acute respiratory failure. These indices may complement conventional imaging and clinical severity scores by providing additional prognostic information. However, the incremental prognostic value of these parameters beyond established scoring systems was not statistically confirmed in our analysis. Given the single-center retrospective design, limited sample size, and exploratory subgroup analyses, the results should be interpreted cautiously. Larger prospective, multicenter studies with external validation are required to confirm these findings and to determine their potential clinical applicability.

## Figures and Tables

**Table 1:** Univariable and multivariable analysis for the first stage.

	Univariable Analysis		Multivariable Analysis	
	OR (95% CI)	*p*	aOR (%95 CI)	*p*
Lymphocyte	0.88 (0.56–1.38)	0.590	1.26 (0.56–2.82)	0.573
Albumin	0.91 (0.84–0.99)	0.030	0.98 (0.89–1.08)	0.714
Urea	1.01 (0.99–1.02)	0.073	1 (0.99–1.02)	0.502
APACHE II	1.14 (1.05–1.23)	0.001	1.08 (0.96–1.22)	0.205
SOFA	1.42 (1.17–1.72)	<0.001	1.34 (0.99–1.81)	0.060
Ventilator days	0.99 (0.97–1.02)	0.728	1 (0.97–1.04)	0.998
Lung LDV (difference) (mL)				
>45	3.27 (1.32–8.10)	0.010	3.24 (0.94–11.11)	0.062
≤45 (R)	1.0	**-**	1.0	-
Lung HDV (difference) (mL)				
>193	2.99 (1.12–8.03)	0.029	4.29 (1.06–17.38)	0.041
≤193 (R)	1.0	**-**	1.0	-
Lung Volume (difference) (mL)				
>246.3	6.13 (2.21–16.97)	<0.001	3.37 (0.85–13.42)	0.085
≤246.3	1.0	**-**	1.0	-

OR: odds ratio, aOR: adjusted odds ratio, CI: confidence interval, R: reference category, APACHE II: Acute Physiology and Chronic Health Evaluation II, SOFA: Sequential Organ Failure Assessment, LDV: low-density volume, and HDV: high-density volume.

**Table 2:** Baseline characteristics for the second stage.

	Total (n = 47)	Survived (n = 18)	Deceased (n = 29)	*p*	d/η^2^/ϕ
Age (years), mean ± SD	64.87 ± 16.05	61.67 ± 20.71	66.86 ± 12.32	0.345	
Male, n (%)	26 (55.3)	8 (44.4)	18 (62.1)	0.379	
Patients requiring IMV, n (%)	40 (85.1)	11 (61.1)	29 (100)	**0.001**	**0.53**
DM, n (%)	14 (29.8)	6 (33.3)	8 (27.6)	0.928	
HT, n (%)	23 (48.9)	7 (38.9)	16 (55.2)	0.432	
CAD, n (%)	9 (19.1)	4 (22.2)	5 (17.2)	0.716	
CKD, n (%)	3 (6.4)	0 (0)	3 (10.3)	NA	
Surgical history, n (%)	11 (23.4)	3 (16.7)	8 (27.6)	0.492	
Solid tumor, n (%)	13 (27.7)	2 (11.1)	11 (37.9)	0.091	
Lymphoma, n (%)	4 (8.5)	0 (0)	4 (13.8)	NA	
Leukemia, n (%)	1 (2.1)	1 (5.6)	0 (0)	NA	
Care-dependent patient, n (%)	11 (23.4)	7 (38.9)	4 (13.8)	0.076	
APACHE II, mean ± SD	22.81 ±7.75	19.06 ± 6.37	25.14 ± 7.69	**0.007**	**0.84**
SOFA, mean ± SD	6.74 ± 2.75	5.39 ± 2.40	7.59 ± 2.65	**0.006**	**0.86**
Time to intubation, median (IQR)	1 (1–1)	1 (1–1)	1 (1–1)	0.506	
ICU length of stay, median (IQR)	8 (3–13)	11 (5–27.5)	8 (3–11)	**0.033**	**0.10**
Ventilator days, median (IQR)	3 (2–10)	2 (0–16.25)	4 (2–9.5)	0.198	
Hospital length of stay, median (IQR)	11 (6–24)	18 (7.5–39)	9 (3–16)	**0.011**	**0.14**

SD: standard deviation, IQR: interquartile range, and NA: not applicable. Independent samples *t*-test, Mann–Whitney U test, and Chi-square test were used for the parameters presented with mean ± SD, median (IQR), and n (%), respectively. IMV: Invasive Mechanical Ventilation, DM: Diabetes Mellitus, HT: Hypertension, CAD: Coronary Artery Disease, CKD: Chronic Kidney Disease, APACHE II: Acute Physiology and Chronic Health Evaluation II, SOFA: Sequential Organ Failure Assessment, and ICU: intensive care unit. d: Effect size for independent samples *t*-test η^2^: Effect size for Mann–Whitney U test. ϕ: Effect size for Chi-square test.

**Table 3:** Quantitative CT indices for the second stage.

	Total (n = 47)	Survived (n = 18)	Deceased (n = 29)	*p*	η^2^
R Lung LDV (mL)	102 (45–394)	92.5 (39–224)	116 (58–435)	0.358	
L Lung LDV (mL)	102 (35–422)	99 (34.25–180.75)	163 (33.5–487)	0.418	
Lung LDV (difference) (mL)	56 (28–174)	36.5 (16.75–77.75)	85 (46–238)	**0.010**	**0.14**
LDV (mL)	242 (112–714)	164 (101.25–517.5)	301 (116.5–839)	0.251	
R Lung MDV (mL)	597 (288–938)	460 (309.75–703.75)	667 (236–963.5)	0.212	
L Lung MDV (mL)	490 (277–755)	475.5 (314–669.75)	603 (276–817.5)	0.654	
Lung MDV (difference) (mL)	268 (132–411)	161 (120.25–246.5)	307 (153.5–525.5)	**0.020**	**0.11**
MDV (mL)	1100 (702–1583)	978.5 (574.5–1434.25)	1303 (771.5–1675.5)	0.347	
R Lung HDV (mL)	557 (439–844)	506 (410.5–724.25)	609 (440–877)	0.615	
L Lung HDV (mL)	487 (303–761)	436.5 (276.5–765)	490 (317–771)	0.887	
Lung HDV (difference) (mL)	186 (93–348)	153 (36.5–202.75)	229 (106–474.5)	**0.029**	**0.10**
HDV (mL)	1100 (805–1463)	899.5 (708.25–1435.25)	1104 (812–1473)	0.555	
R Lung Tissue V (mL)	682 (335–1264)	507 (350.75–1075.5)	841 (306.5–1377.5)	0.216	
L Lung Tissue V (mL)	676 (312–1192)	605 (394.75–790)	809 (311–1260)	0.470	
Lung Tissue V (difference) (mL)	344 (160–560)	182.5 (149–289.25)	474 (270–821.5)	**0.011**	**0.14**
Lung Tissue V (mL)	1419 (824–2269)	1158 (675.75–2030)	1518 (916–2760)	0.225	
R L LD Index (%)	0.18 (0.12–0.31)	0.16 (0.14–0.27)	0.2 (0.11–0.32)	0.622	
L L LD Index (%)	0.21 (0.13–0.31)	0.18 (0.13–0.25)	0.22 (0.13–0.33)	0.303	
LD Index (difference)	0.03 (0.01–0.06)	0.03 (0.01–0.03)	0.03 (0.01–0.09)	0.224	
LD Index (%)	0.21 (0.13–0.3)	0.16 (0.14–0.26)	0.24 (0.13–0.33)	0.259	
R L PD15 (g/L)	68 (28–89)	73 (30.75–86)	61 (27.5–90.5)	0.827	
L L PD15 (g/L)	55 (29–87)	69.5 (41.25–88.75)	53 (28–86.5)	0.399	
PD15 (difference)	7 (2–21)	6.5 (4–13.25)	7 (2–26)	0.677	
PD15 (g/L)	55 (27–84)	70.5 (43–82)	48 (25–84.5)	0.375	
R Up Lung LDI (%)	0.2 (0.12–0.3)	0.16 (0.12–0.26)	0.21 (0.12–0.33)	0.622	
L Up Lung LDI (%)	0.21 (0.14–0.31)	0.18 (0.15–0.31)	0.22 (0.14–0.35)	0.443	
Up Lung LDI (difference)	0.03 (0.01–0.07)	0.03 (0.01–0.05)	0.02 (0.01–0.1)	0.659	
Up Lung LDI (%)	0.2 (0.13–0.31)	0.16 (0.12–0.25)	0.22 (0.13–0.33)	0.193	
R Lo. Lung LDI (%)	0.17 (0.1–0.34)	0.17 (0.14–0.34)	0.18 (0.1–0.33)	0.693	
L Lo. Lung LDI (%)	0.18 (0.1–0.3)	0.17 (0.13–0.26)	0.21 (0.09–0.34)	0.443	
Lo.Lung LDI (difference)	0.03 (0.01–0.08)	0.03 (0.01–0.08)	0.04 (0.01–0.1)	0.496	
Lo.Lung LDI (%)	0.19 (0.14–0.32)	0.17 (0.14–0.28)	0.21 (0.12–0.37)	0.477	
R L Up Low Ratio	0.97 (0.74–1.23)	0.84 (0.74–1.03)	1 (0.73–1.44)	0.120	
L L Up Low Ratio	1.13 (0.89–1.44)	1.09 (0.89–1.26)	1.26 (0.89–1.81)	0.165	
Up Low Ratio (difference)	0.34 (0.16–0.58)	0.31 (0.19–0.46)	0.37 (0.16–0.83)	0.734	
Up Low Ratio	1.03 (0.83–1.4)	0.96 (0.82–1.13)	1.13 (0.85–1.48)	0.120	
R T Lung Volume (mL)	1568.3 (1027.2–2026)	1200.4 (1008.4–1649.8)	1653.3 (947.0–2276.2)	0.175	
L T Lung Volume (mL)	1373.0 (682.5–1796.9)	1205.6 (859.0–1599.0)	1427 (608.0–1911.4)	0.498	
Lung Volume (difference) (mL)	550.1 (283.5–1027.1)	284.9 (93.8–498.6)	688.0 (399.7–1167.4)	**<0.001**	**0.26**
Airways (mL)	30.72 (17.22–43.36)	31.68 (16.21–56.32)	29.87 (18.31–41.94)	0.793	
Total Lung Volume (mL)	2819.8 (2160.7–3909.6)	2469.76(2123.9–3294.4)	2950.1 (2141.7–4209.0)	0.347	
Mean HU R Lung (HU)	−635 (−741-(−567))	−625 (−705.75-(−526.75))	−654 (−769-(−571.5))	0.293	
Mean HU L Lung (HU)	−689 (−739-(−581))	−671.5 (−738.25-(−548.5))	−689 (−751-(−583))	0.827	
Mean HU Lung (difference)	53 (27–102)	37 (21–99.25)	55 (28.5–148)	0.364	
Mean HU Airways (HU)	−967 (−981-(−948))	−962.5 (−973.75-(−946.5))	−973 (−987-(−948.5))	0.110	

Parameters are presented with median (IQR). Mann–Whitney U test was used. R Lung LDV: right lung low-density volume, L Lung LDV: left lung low-density volume, LDV: low-density volume, R Lung MDV: right lung medium-density volume, L Lung MDV: left lung medium-density volume, Lung MDV: lung medium density volume, R Lung HDV: right lung high-density volume, L Lung HDV: left lung high-density volume, Lung HDV: lung high-density volume, R Lung Tissue V: right lung tissue volume, L Lung tissue V: left lung tissue volume, Lung tissue V: lung tissue volume, R L LD index: right lung low-density index, L L LDindex: left lung low-density index, LD index: low-density index, R L PD15: right lung 15th percentile density, L L PD15: left lung 15th percentile density, PD15: 15th percentile density, R L Up Lung LDI: right upper lung low-density index, Up Lung LDI: upper lung low-density volume, R Lo. Lung LDI: right lower lung low-density index, L Lo. Lung LDI: left lower lung density index, Lo.Lung LDI: lower lung low-density index, R L Up Low Ratio: right lung upper lower ratio, L L Up Low Ratio: left lung upper lower ratio, Up Low Ratio: upper lower ratio, R T Lung Volume: right total lung volume, L T Lung Volume: left total lung volume, Mean HU R Lung: mean HU right lung, and Mean HU L Lung: mean HU left lung. η^2^: Effect size for Mann–Whitney U test.

**Table 4:** Univariable and multivariable analysis for the second stage.

	Univariable Analysis		Multivariable Analysis	
	OR (95% CI)	*p*	aOR (95%CI)	*p*
Albumin	0.87 (0.77–0.99)	**0.031**	**1.03 (0.85–1.2)**	**0.79**
Lymphocyte	0.91 (0.53–1.55)	0.727	0.94 (0.40–2.2)	0.89
APACHE II	1.13 (1.03–1.25)	**0.014**	**1.10 (0.92–1.32)**	**0.27**
SOFA	1.41 (1.08–1.84)	**0.012**	**1.37 (0.80–2.37)**	**0.26**
ICU length of stay	0.93 (0.87–0.99)	**0.034**	**0.95 (0.88–1.03)**	**0.22**
Lung MDV (difference) (mL)				
>219	9.19 (2.32–36.43)	**0.002**	**9.30 (1.23–70.30)**	**0.03**
≤219 (R)	1.0	**-**		
Lung HDV (difference) (mL)				
>193	5.73 (1.5–21.89)	**0.011**	**10.85 (1.32–89.28)**	**0.02**
≤193 (R)	1.0	**-**		

OR: odds ratio, CI: confidence interval, aOR: adjusted odds ratio, R: reference category, APACHE II: Acute Physiology and Chronic Health Evaluation II, SOFA: Sequential Organ Failure Assessment, MDV: medium-density volume, HDV: high-density volume, and ICU length of stay: intensive care unit length of stay.

**Table 5:** AUC values for Stage I and Stage II.

		Cut-Off Value	AUC (95% CI)	Sensitivity	Specificity	*p*
**First stage**	**Lung LDV (difference)** (mL)	>45	0.653 (0.544–0.751)	63.16	65.62	**0.010**
	**Lung HDV(difference)** (mL)	>193	0.626 (0.517–0.726)	45.61	78.12	**0.036**
	**Lung Volume (difference)** (mL)	>246	0.684 (0.576–0.778)	85.96	50	**0.002**
**Second stage**	**Lung LDV (difference)** (mL)	>45	0.725 (0.575–0.845)	75.86	66.67	**0.003**
	**Lung MDV (difference)** (mL)	>219	0.703 (0.552–0.827)	72.41	77.78	**0.012**
	**Lung HDV (difference)** (mL)	>193	0.691 (0.539–0.817)	62.07	77.78	**0.014**
	**Lung V (difference)** (mL)	>264	0.722 (0.572–0.843)	75.86	77.78	**0.006**
	**Lung Volume (difference)** (mL)	>550	0.808 (0.667–0.908)	68.97	83.33	**<0.001**

Lung LDV: lung low-density volume, Lung HDV: lung high-density volume, Lung V: lung tissue volume, and Lung Volume: total lung volume.

## Data Availability

The dataset will be shared upon request via e-mail.

## Supplementary Materials

The following are available online at https://www.mdpi.com/article/10.3390/diagnostics16050685/s1, Table S1: Baseline Characteristics for First Stage, Table S2: Etiological Agents of Pneumonia, Table S3: Laboratory Values for First Stage, Table S4: Quantitative CT Indices for First Stage, Table S5: Etiological Agents of Pneumonia for Second Stage, Table S6: Laboratory Values for second stage.
