# Supplementary material for: Evaluation of Quantitative Computed Tomography Indices in Patients with Pneumonia and Acute Respiratory Failure in the Intensive Care Unit (ICU)
Source: Diagnostics (Basel). 2026 Feb 26;16(5):685. doi: 10.3390/diagnostics16050685 (PMC12984187; doi:10.3390/diagnostics16050685)
Supplement: Supplementary file 1 [file diagnostics-16-00685-s001.zip › Suplemantary Table 1 Baseline Characteristics .pdf]

Suplemantary Table S1 Baseline Characteristics for First Stage

	**Total (n=89)**	**Survived(n=32)**	**Deceased (n=57)**	**p**
Age (years), mean ± SD	66.78 ± 15.03	65.38 ± 17.57	67.56 ± 13.51	0.513
Male, n (%)	52 (58.4)	19 (59.4)	33 (57.9)	1.0
Patients requiring IMV, n (%)	72 (80.9)	15 (46.9)	57 (100)	**<0.001**
HT, n (%)	46 (51.7)	14 (43.8)	32 (56.1)	0.367
CAD, n (%)	20 (22.5)	8 (25)	12 (21.1)	0.870
CKD, n (%)	10 (11.2)	0	10 (17.5)	**0.012**
Surgical history, n (%)	24 (27)	9 (28.1)	15 (26.3)	1.0
Solid tumor, n (%)	23 (25.8)	5 (15.6)	18 (31.6)	0.162
Lymphoma, n (%)	4 (4.5)	0	4 (7)	NA
Leukemia, n (%)	2 (2.2)	1 (3.1)	1 (1.8)	NA
Care-dependent patient, n (%)	20 (22.5)	11 (34.4)	9 (15.8)	0.080
APACHEII, mean ± SD	22.82 ± 7.27	19.13 ± 5.89	24.89 ± 7.18	**<0.001**
SOFA, median (IQR)	6 (4-9)	5 (3-6.75)	8 (5.5-10)	**<0.001**
Time to intubation, median (IQR)	1 (1-1)	1 (1-1)	1 (1-1.5)	0.061
ICU length of stay, median (IQR)	8 (5-13)	8 (5-11)	8 (4-13)	0.777
Ventilator days, median (IQR)	4 (1-10)	0.5 (0-6)	6 (3-11)	**<0.001**
Hospital length of stay, median (IQR)	12 (7.5-18)	13 (8.25-29.25)	10 (7-16.5)	0.102

SD: Standard deviation, IQR: Interquartile range, NA: Not applicable

Independent Samples t-test, Mann-Whitney U test, and Chi-square test were used for the parameters presented with mean ± SD, median (IQR), and n (%), respectively. IMV:Invasive Mechanical Ventilation, DM: Diabetes Mellitus, HT: Hypertension, CAD: Coronary Artery Disease, CKD: Chronic Kidney Disease, APACHE II: Acute Physiology And Chronic Health Evaluation II, SOFA: Sequential Organ Failure Assessment, ICU: Intensive Care Unit
