# Supplementary material for: Evaluation of Quantitative Computed Tomography Indices in Patients with Pneumonia and Acute Respiratory Failure in the Intensive Care Unit (ICU)
Source: Diagnostics (Basel). 2026 Feb 26;16(5):685. doi: 10.3390/diagnostics16050685 (PMC12984187; doi:10.3390/diagnostics16050685)
Supplement: Supplementary file 1 [file diagnostics-16-00685-s001.zip › Suplemantary Table 2 Etiological Agents of Pneumonia first stage.pdf]

Suplemantary Table S2 Etiological Agents of Pneumonia

	**Total (n=89)**	**Survived (n=32)**	**Deceased (n=57)**	**p**
Bacteria n (%)	29 (32.6)	6 (18.8)	23 (40.4)	0.064
Viral, n (%)	24 (27)	9 (28.1)	15 (26.3)	1.0
Fungal, n (%)	5 (5.6)	3 (9.4)	2 (3.5)	NA

Chi-square test was used.
