# Supplementary material for: Evaluation of Quantitative Computed Tomography Indices in Patients with Pneumonia and Acute Respiratory Failure in the Intensive Care Unit (ICU)
Source: Diagnostics (Basel). 2026 Feb 26;16(5):685. doi: 10.3390/diagnostics16050685 (PMC12984187; doi:10.3390/diagnostics16050685)
Supplement: Supplementary file 1 [file diagnostics-16-00685-s001.zip › Suplemantary Table 3 Laboratory Values.pdf]

Suplemantary Table S3 Laboratory Values for First Stage

	**Total (n=89)**	**Yaşıyor (n=32)**	**Ex (n=57)**	**p**
Hemoglobin (g/dL), mean ± SD	10.79 ± 2.18	11.36 ± 2.01	10.47 ± 2.22	0.065
Leukocyte (10^3/µL), median (IQR)	10 (6.3-16.2)	9.85 (6.95-14.23)	10.4 (4.9-18.75)	0.784
Lymphocyte (10^3/µL), median (IQR)	0.7 (0.33-1.2)	0.94 (0.61-1.2)	0.46 (0.26-1.15)	**0.010**
Platelet (10^3/µL), median (IQR)	222 (139-323.5)	263 (165.25-320.5)	206 (102.5-323.5)	0.226
C-RP, median (IQR)	149 (51-255)	116 (20.5-238)	184 (66.5-278)	0.132
Albumin (g/dL), mean ± SD	29.48 ± 6.21	31.44 ± 7.15	28.37± 5.36	**0.025**
LDH, median (IQR)	325 (221.5-471)	267.5 (206.75-375.75)	347 (232-557)	0.107
AST, median (IQR)	27 (17.5-63)	23.5 (17.25-47)	31 (17.5-68.5)	0.412
ALT, median (IQR)	22.5 (12.25-35.75)	19 (11-27)	25 (13-39.5)	0.073
Urea, median (IQR)	55 (34.25-89)	46 (26.25-71.25)	62 (37.25-98.5)	**0.032**
Creatinine, median (IQR)	0.89 (0.62-1.43)	0.77 (0.6-1.28)	1 (0.64-1.7)	0.192
D-Dimer, median (IQR)	2.4 (1.15-5.05)	2.4 (1.33-7.5)	2.45 (0.86-5.08)	0.344
PT, median (IQR)	13 (11-15)	13 (11-14.75)	14 (11-15)	0.256
aPTT, median (IQR)	26 (22-30.75)	25.5 (22-28.75)	27 (23-32)	0.167
pH, median (IQR)	7.4 (7.33-7.46)	7.42 (7.32-7.47)	7.4 (7.33-7.46)	0.439
pO2, median (IQR)	75 (60-108.5)	71 (60-83)	85 (61.5-112)	0.145
pCO2, median (IQR)	35 (29-45.5)	35.5 (30-46.75)	35 (28-45)	0.765
Lactate, median (IQR)	1.6 (1.1-2.4)	1.3 (1.1-2.2)	1.6 (1.1-2.5)	0.271
BE, median (IQR)	-1.3 (-3.85-1.3)	-1.4 (-3.18-2.48)	-1 (-4.65-1.15)	0.439

SD: Standard deviation, IQR: Interquartile range

Independent Samples t-test and Mann-Whitney U test were used for the parameters presented with mean ± SD and median (IQR), respectively

BE, base excess; PT, prothrombin time; aPTT, activated partial thromboplastin time; LDH, lactate dehydrogenase; AST, aspartate aminotransferase; ALT, alanine aminotransferase; CRP, C-reactive protein
