# Supplementary material for: Evaluation of Quantitative Computed Tomography Indices in Patients with Pneumonia and Acute Respiratory Failure in the Intensive Care Unit (ICU)
Source: Diagnostics (Basel). 2026 Feb 26;16(5):685. doi: 10.3390/diagnostics16050685 (PMC12984187; doi:10.3390/diagnostics16050685)
Supplement: Supplementary file 1 [file diagnostics-16-00685-s001.zip › Suplemantary Table 4 Quantitative CT Indices for First Stage.pdf]

Suplemantary Table S4 Quantitative CT Indices for First Stage

	**Total (n=89)**	**Yaşıyor (n=32)**	**Ex (n=57)**	**p**
R LungLDV(ml)	136 (54-401)	109.5 (49.75-306.25)	139 (58-438.5)	0.570
L LungLDV (ml)	115 (37-378.5)	108.5 (66-244.75)	140 (31-414.5)	0.878
Lung LDV (difference)	53 (25.5-155.5)	33.5 (17.25-89.5)	78 (31-174)	**0.017**
LDV (ml)	301 (126-732.5)	200.5 (133.25-530.5)	337 (116.5-820)	0.460
R Lung MDV (ml)	647 (313-956.5)	555.5 (318.75-883.5)	667 (279.5-966.5)	0.700
L Lung MDV(ml)	490 (276-786)	493 (340.25-750)	490 (242.5-837.5)	0.814
Lung MDV (difference)	182 (92.5-372.5)	146 (70.5-329)	266 (103-385)	0.140
MDV (ml)	1136 (678-1693)	1162 (688.5-1676.5)	1100 (617-1703.5)	0.918
R Lung HDV (ml)	603 (450-853)	608.5 (486.25-853.5)	600 (440-852.5)	0.629
L Lung HDV (ml)	493 (343.5-737.5)	577.5 (346.5-760.5)	454 (343.5-691)	0.313
Lung HDV (difference)	141 (55-249.5)	112.5 (35-191.25)	168 (57-327)	**0.049**
HDV (ml)	1142 (817.5-1461)	1256.5 (808.5-1568)	1060 (822-1449)	0.467
R Lung Tissue V (ml)	758 (367-1356.5)	674 (386.25-1245)	853 (336.5-1444.5)	0.541
L Lung Tissue V (ml)	695 (311-1195.5)	662 (439.75-1000)	711 (276-1253)	0.993
Lung Tissue V (difference)	236 (108-520)	165 (69.75-411.5)	331 (110.5-544)	0.112
Lung Tissue V (ml)	1462 (817.5-2596.5)	1373.5 (787-2194.75)	1518 (817.5-2785.5)	0.647
R L LD index (%)	0.21 (0.12-0.32)	0.21 (0.12-0.28)	0.22 (0.11-0.34)	0.549
L L LDindex (%)	0.22 (0.12-0.31)	0.22 (0.14-0.28)	0.22 (0.11-0.32)	0.650
LD index (difference)	0.03 (0.01-0.07)	0.03 (0.01-0.05)	0.03 (0.01-0.08)	0.354
LD index (%)	0.22 (0.13-0.31)	0.22 (0.14-0.27)	0.24 (0.12-0.34)	0.311
R L PD15 (g/L)	51 (28.5-90.5)	53 (35.25-88.5)	50 (27.5-94.5)	0.781
L L PD15 (g/L)	53 (29-89)	53.5 (33.75-81.5)	48 (26.5-91.5)	0.781
PD15 (difference)	8 (4-19.5)	7 (5-14)	8 (4-25)	0.546
PD15 (g/L)	51 (26-84.5)	52 (36-81)	48 (25-87.5)	0.414
R Up Lung LDI (%)	0.21 (0.11-0.32)	0.2 (0.12-0.29)	0.21 (0.1-0.34)	0.831
L Up Lung LDI (%)	0.22 (0.13-0.36)	0.21 (0.15-0.34)	0.22 (0.12-0.36)	0.881
Up Lung LDI (difference)	0.03 (0.01-0.08)	0.04 (0.01-0.07)	0.02 (0.01-0.09)	0.744
Up Lung LDI (%)	0.21 (0.13-0.32)	0.2 (0.13-0.3)	0.22 (0.12-0.33)	0.505
R Lo. Lung LDI (%)	0.19 (0.1-0.33)	0.18 (0.11-0.32)	0.21 (0.1-0.34)	0.834
L Lo. Lung LDI (%)	0.2 (0.11-0.29)	0.19 (0.13-0.25)	0.2 (0.1-0.32)	0.635
Lo.Lung LDI (difference)	0.03 (0.01-0.08)	0.03 (0.01-0.08)	0.03 (0.01-0.08)	0.780
Lo.Lung LDI (%)	0.2 (0.12-0.32)	0.2 (0.14-0.28)	0.21 (0.12-0.37)	0.392
R L Up Low Ratio	0.97 (0.73-1.29)	0.89 (0.78-1.26)	1 (0.71-1.35)	0.635
L L Up Low Ratio	1.16 (0.88-1.5)	1.17 (0.93-1.31)	1.13 (0.72-1.68)	0.513
Up Low Ratio (difference)	0.29 (0.14-0.51)	0.31 (0.16-0.51)	0.29 (0.13-0.51)	0.854
Up Low Ratio	1.03 (0.83-1.43)	1.02 (0.84-1.25)	1.05 (0.81-1.5)	0.611
R T Lung Volume (ml)	1630.2 (1077.5-2094.8)	1611.0 (1061.8-1999.6)	1653.3 (1098.6-2213.6)	0.578
L T Lung Volume (ml)	1367.0 (861.9-1819.7)	1293.2 (1012.5-1720.0)	1373.0 (750.4-1911.4)	0.837
Lung Volume (difference)	416.6 (231.8-696.1)	259.49(136.9-542.0)	505 (291.1-970.7)	**0.004**
Airways (ml)	30.7 (17.9-43.3)	33.6 (17.3-51.5)	27.7 (18.3-43.0)	0.584
Total Lung Volume (ml)	2950.1 (2220.5-3919.19	2971.5 (2308.3-3804.6)	2950.1 (2141.7-4088)	0.939
Mean HU R Lung (HU)	-643 (-747-(-553.5))	-637 (-733.5-(-541.75))	-657 (-769-(-559))	0.278
Mean HU L Lung (HU)	-662 (-743.5-(-564.5))	-658.5 (-736.5-(-557.75))	-662 (-758.5-(-564.5))	0.794
Mean HU Lung (difference)	36 (15-79.5)	36.5 (12.25-74.5)	36 (15-85.5)	0.811
Mean HU Airways (HU)	-968 (-982-(-948.5))	-964.5 (-974.75-(-955.5))	-971 (-987-(-944))	0.245

Parameters were presented with median (IQR).Mann-Whitney U test was used. R Lung LDV: Right lung low density volume, L Lung LDV: Left lung low density volume, LDV: Low density volume, R Lung MDV: Right lung medium density volume, L Lung MDV: Left lung medium density volume, Lung MDV: Lung medium density volume, R Lung HDV: Right lung high density volume, L Lung HDV: Left lung high density volume, Lung HDV: Lung high density volume, R Lung P V: Right lung tissue volüme, L Lung P V: Left Lung tissue volüme, Lung P V: Lung tissue volüme, R L LD index: Right lung low density index, L L LDindex: Left lung low density index, LD index: Low density index, R L PD15: Right lung 15th Percentile density, L L PD15: Left lung 15th Percentile Density, PD15: 15th Percentile density, R L Up Lung LDI: Right upper lung low density index, Up Lung LDI: Upper lung low density volume, R Lo. Lung LDI: Right lower lung low density index, L Lo. Lung LDI: Left lower lung density index, Lo.Lung LDI: Lower lung low density index, R L Up Low Ratio: Right lung upper lower ratio, L L Up Low Ratio: Left lung upper lower ratio, Up Low Ratio: Upper lower ratio, R T Lung Volume: Right total lung volume, L T Lung Volume: Left total lung volume, Mean HU R Lung: Mean HU Right Lung, Mean HU L Lung: Mean HU Left Lung
