# Supplementary material for: Evaluation of Quantitative Computed Tomography Indices in Patients with Pneumonia and Acute Respiratory Failure in the Intensive Care Unit (ICU)
Source: Diagnostics (Basel). 2026 Feb 26;16(5):685. doi: 10.3390/diagnostics16050685 (PMC12984187; doi:10.3390/diagnostics16050685)
Supplement: Supplementary file 1 [file diagnostics-16-00685-s001.zip › Suplemantary Table 5 Etiological Agents of Pneumonia second stage.pdf]

Suplemantary Table S5 Etiological Agents of Pneumonia for Second Stage

	**Total (n=47)**	**Survived (n=18)**	**Deceased (n=29)**	**p**
Bacteria n (%)	18 (38.3)	4 (22.2)	14 (48.3)	0.140
Viral, n (%)	12 (25.5)	7 (38.9)	5 (17.2)	0.168
Fungal, n (%)	4 (8.5)	3 (16.7)	1 (3.4)	NA

Chi-square test was used
