# Supplementary material for: Evaluation of Quantitative Computed Tomography Indices in Patients with Pneumonia and Acute Respiratory Failure in the Intensive Care Unit (ICU)
Source: Diagnostics (Basel). 2026 Feb 26;16(5):685. doi: 10.3390/diagnostics16050685 (PMC12984187; doi:10.3390/diagnostics16050685)
Supplement: Supplementary file 1 [file diagnostics-16-00685-s001.zip › Suplemantary Table 6 Laboratory Values for second stage.pdf]

Suplemantary Table S6 Laboratory Values for second stage

	**Total (n=47)**	**Yaşıyor (n=18)**	**Ex (n=29)**	**p**
Hemoglobin (g/dL), mean ± SD	10.58 ± 2.0	11.07 ± 2.16	10.27 ± 1.87	0.188
Leukocyte ($10^3/\mu L$), median (IQR)	10.2 (5.3-20)	10.1 (8.08-14.9)	10.4 (3.38-21.75)	0.870
Lymphocyte ($10^3/\mu L$), median (IQR)	0.7 (0.23-1.2)	0.94 (0.6-1.28)	0.46 (0.15-1.2)	0.043
Platelet ($10^3/\mu L$), median (IQR)	219 (108-319)	282.5 (150-331.75)	180 (80.5-292)	0.084
C-RP, median (IQR)	177 (58-284)	127.5 (18.75-255.25)	184 (74-296.5)	0.325
Albumin (g/dL), mean ± SD	29.23 ± 5.72	31.61 ± 5.47	27.75 ± 5.45	**0.023**
LDH, median (IQR)	329 (230-455)	307.5 (223.75-419)	347 (217.5-459)	0.511
AST, median (IQR)	26 (17-55)	23.5 (17-42)	33 (17.5-68)	0.387
ALT, median (IQR)	21 (12-35)	15.5 (10-27.75)	29 (13-39.5)	**0.021**
Urea, median (IQR)	59 (36-89.25)	52.5 (22.5-88)	61 (42.5-89.75)	0.280
Creatinine, median (IQR)	0.81 (0.6-1.36)	0.77 (0.57-1.42)	0.87 (0.61-1.42)	0.577
D-Dimer, median (IQR)	3.05 (1.47-5.08)	2.85 (1.47-4.18)	3.4 (1.42-6.15)	0.798
PT, median (IQR)	12.5 (11-15.25)	12.5 (11-16)	13 (11-15)	0.609
aPTT, median (IQR)	27 (22-32)	27.5 (22-30.75)	26.5 (22-32)	0.676
pH, median (IQR)	7.44 (7.34-7.48)	7.44 (7.36-7.49)	7.44 (7.29-7.47)	0.411
pO2, median (IQR)	74 (59-110)	71.5 (58.25-88.75)	78 (58.5-111)	0.437
pCO2, median (IQR)	35 (30-45)	35 (28.75-38)	35 (30-56)	0.381
Lactate, median (IQR)	1.6 (1.1-2.4)	1.55 (1.1-2.25)	1.7 (1.2-3.05)	0.324
BE, median (IQR)	-0.6 (-3.8-2.4)	0.25 (-3.35-2.83)	-0.9 (-4.2-1.55)	0.615

SD: Standard deviation, IQR: Interquartile range

Independent Samples t-test and Mann-Whitney U test were used for the parameters presented with mean ± SD and median (IQR), respectively

BE, base excess; PT, prothrombin time; aPTT, activated partial thromboplastin time; LDH, lactate dehydrogenase; AST, aspartate aminotransferase; ALT, alanine aminotransferase; CRP, C-reactive protein
